# Retraction Note: Long noncoding RNA SNHG1 promotes TERT expression by sponging miR-18b-5p in breast cancer

**DOI:** 10.1186/s13578-023-00984-x

**Published:** 2023-02-19

**Authors:** Yujuan Kang, Lin Wan, Qin Wang, Yanling Yin, Jiena Liu, Lei Liu, Hao Wu, Lei Zhang, Xin Zhang, Shouping Xu, Da Pang

**Affiliations:** 1 Department of Breast Surgery, Harbin Medical University Cancer Hospital, Harbin, 150040 China; 2 Heilongjiang Academy of Medical Sciences, Harbin, China

**Retraction Note: Cell & Bioscience (2021) 11:169** **https://doi.org/10.1186/s13578-021-00675-5**

The Editors have retracted this article at the request of the authors because several irregularities have been identified in the figures. Specifically:

- The NC and SiSNHG1#1 panels at 0 h for MDA-MB-468 cells in Fig. 2e contain overlapping data.
- The SiSNHG1/MDA-MB-468 panel in Fig. 5l is a duplicate of the SiSNHG1#2/MDA-MB-468 panel in Fig. 2f. There also appears to be an overlap between the panels for NC/MDA-MB-468 between these two figures.
- The panel for miR-18b-3p mimics in MDA-MB-231 cells in Fig. 4l contains overlap with the SiSNHG1#1 panel for MDA-MB-231 cells in Fig. 2f.

Additionally, concerns have been raised regarding ethics approval for the human part of the study, as well as mouse tumour ulceration in Fig. 2k. In view of the number of errors, the article is being retracted.

All authors agree to this retraction.

